# Shaping innovations in long-term care for stroke survivors with multimorbidity through stakeholder engagement

**DOI:** 10.1371/journal.pone.0177102

**Published:** 2017-05-05

**Authors:** Euan Sadler, Talya Porat, Iain Marshall, Uy Hoang, Vasa Curcin, Charles D. A. Wolfe, Christopher McKevitt

**Affiliations:** 1 Division of Health and Social Care Research, Faculty of Life Sciences and Medicine, King’s College London, London, United Kingdom; 2 King’s Improvement Science, Centre for Implementation Science, Health Service and Population Research Department, Institute of Psychiatry, Psychology and Neuroscience, King’s College London, London, United Kingdom; 3 National Institute for Health Research Collaboration for Leadership in Applied Health Research and Care South London, King’s College Hospital NHS Foundation Trust and King’s College London, London, United Kingdom; 4 National Institute for Health Research Biomedical Research Centre at Guy's and St Thomas' NHS Foundation Trust and King's College London, London, United Kingdom; Centro de Neurociencias de Cuba, CUBA

## Abstract

**Background:**

Stroke, like many long-term conditions, tends to be managed in isolation of its associated risk factors and multimorbidity. With increasing access to clinical and research data there is the potential to combine data from a variety of sources to inform interventions to improve healthcare. A ‘Learning Health System’ (LHS) is an innovative model of care which transforms integrated data into knowledge to improve healthcare. The objective of this study is to develop a process of engaging stakeholders in the use of clinical and research data to co-produce potential solutions, informed by a LHS, to improve long-term care for stroke survivors with multimorbidity.

**Methods:**

We used a stakeholder engagement study design informed by co-production principles to engage stakeholders, including service users, carers, general practitioners and other health and social care professionals, service managers, commissioners of services, policy makers, third sector representatives and researchers. Over a 10 month period we used a range of methods including stakeholder group meetings, focus groups, nominal group techniques (priority setting and consensus building) and interviews. Qualitative data were recorded, transcribed and analysed thematically.

**Results:**

37 participants took part in the study. The concept of how data might drive intervention development was difficult to convey and understand. The engagement process led to four priority areas for needs for data and information being identified by stakeholders: 1) improving continuity of care; 2) improving management of mental health consequences; 3) better access to health and social care; and 4) targeting multiple risk factors. These priorities informed preliminary design interventions. The final choice of intervention was agreed by consensus, informed by consideration of the gap in evidence and local service provision, and availability of robust data. This shaped a co-produced decision support tool to improve secondary prevention after stroke for further development.

**Conclusions:**

Stakeholder engagement to identify data-driven solutions is feasible but requires resources. While a number of potential interventions were identified, the final choice rested not just on stakeholder priorities but also on data availability. Further work is required to evaluate the impact and implementation of data-driven interventions for long-term stroke survivors.

## Introduction

### Impact of stroke

Stroke is increasingly recognised as a long-term condition [1–3], yet long-term stroke care is characterised by a lack of continuity of care and inequalities in access to services [4]. Existing interventions targeting specific aspects of long-term need have shown, at best, limited success [5]. These include interventions to improve pharmacological and psychotherapeutic interventions to treat depression [6]; information provision interventions [7]; secondary prevention behavioural interventions [8, 9]; and clinical management of risk factors to reduce stroke recurrence [10–11]. In terms of the latter, in the United Kingdom (UK) recent guidelines for stroke include the prevention and management of stroke recurrence [12], which report on the evidence base of interventions to reduce risk factors associated with specific behaviours and health conditions. However, the evidence base for interventions to address multiple risk factors to reduce secondary prevention in the long-term after stroke is limited. Interventions to address multiple needs have also reported limited or no benefit, including assessment and signposting to available services [7]; liaison worker interventions [13] and self-management programmes [14–18]. One possible reason for this limited effectiveness is that, being professionally designed, interventions addressing long-term needs after stroke may not match service user priorities [19].

Stroke survivors commonly experience multimorbidity, defined as two or more co-existent long-term conditions [20], yet there is little research investigating the challenges facing stroke survivors with multimorbidity. In the UK, the South London Stroke Register (SLSR), a long-standing population register covering an ethnically diverse inner city region [21], estimates that approximately half of stroke survivors report at least two other long-term conditions, such as diabetes, hypertension, atrial fibrillation, depression and cognitive impairment [22]. This is significant since multimorbidity is associated with higher levels of disability [23], reduced quality of life [24], and higher health care use and costs [25]. Challenges facing people with multimorbidity include difficulties prioritising one condition over another [26], management of multiple medication regimes [27], and significant burden on carers [27]. General Practitioners (GPs) and other health care professionals also face complex decision making and report difficulties in the clinical management of people with multimorbidity [28, 29]. Currently there is a tendency to manage stroke in isolation, but the growing attention to multimorbidity needs investigation in the case of the stroke population who have high levels of multimorbidity and associated long-term needs.

Given that the overwhelming majority of published trials and clinical guidelines focus on a selected group of patients with single conditions, what constitutes good management in patients with multimorbidity is ill-defined [30, 31]. A recent Cochrane Review found that interventions to support people with multimorbidity are limited, but there is some evidence that targeting specific risk factors such as depression and functional difficulties improves health outcomes [32]. However, it is not currently known what interventions best meet the long-term needs of stroke survivors with multimorbidity, in a cost effective manner.

### A learning health system

To improve long-term stroke care, health users, systems and services require high quality information to plan and commission care, address unmet long-term need and inequality, and guide best practice for individual patients and the stroke population. The challenges are in supporting the infrastructure needed to manage information from a variety of sources for a variety of stakeholders as well as the development of effective models of care and clinical decision support [33, 34].

One potential solution is a ‘Learning Health System’ (LHS), an innovative model of care originally developed in the USA [35], that treats every participant in the health system (e.g. clinician, patient, commissioner, researcher) as a producer and consumer of data. A LHS relates to ‘the cycle of turning health care data into knowledge, translating that knowledge into practice, and creating new data by means of advanced information technology' (p.54) [36]. It combines routinely collected patient information from a variety of sources, which are anonymised for research purposes to produce a better picture of a local population’s needs, to inform interventions to improve healthcare. A LHS has the potential to improve healthcare for people with long-term conditions by informing interventions integrated with Electronic Health Record (EHR) systems, to deliver recommendations and simultaneously capture additional data back into the system, in order to improve predictive models that the tools are based on. At the centre of a LHS ethos is routine capture, transformation and dissemination of data and knowledge, with various uses, including clinical studies, quality improvement initiatives and decision support, constructed on top of specific routes that the data is taking through the system [37]. Motivating the current study, the opportunity to develop a LHS knowledge base to improve long-term stroke care arose when research data from the SLSR [21] was linked with primary care data from an existing patient record database of local general practices in South London, Lambeth Datanet (LDN), that was geographically coterminous with the SLSR, and provides more detailed phenotypic data on patients and their provision of care.

### A co-production approach to improve healthcare

A LHS potentially offers a real time solution to improve long-term stroke care, but there is a need to incorporate both professional and service user priorities based on data available from multiple sources to improve care and to identify gaps that require attention. A limited number of studies have shown that engaging a range of stakeholders to develop a LHS and integrated clinical decision support systems improve processes of care and outcomes in other long-term conditions [38, 39], but this has not been examined in the context of stroke.

One stakeholder engagement approach which is receiving growing attention in health research is co-production. This approach aims to engage providers and users of services to work collaboratively to improve health and care services, making use of their different experiences, assets and resources to develop, evaluate and implement services to improve outcomes [40–45]. Co-production has its roots in ‘engaged scholarship’ which considers research a collective activity, and is increasingly being used as an effective way to ensure research impact [45]. The value of using a co-production approach in health research has been realised in terms of improving health and social care for frail older people and those with long-term conditions and resultant disabilities [46, 47].

There is currently a lack of consensus about what is meant by co-production and the methods used in co-production research [48]. However, a number of common principles have been identified [43–45]. For example, Heaton and colleagues [45] propose five main principles of co-production: 1) service users as active agents; 2) equal partnership working between service users and professionals; 3) collaboration, reciprocity and mutuality between service users and professionals; 4) potential to transform service design through increased service user participation; and 5) service user participation enabled and supported by networks and organisations. The literature also recognises different ‘forms’ of co-production. For example, Bovaird [49] proposes that co-production approaches range on a continuum from ‘traditional professional service provision with user consultation’ to ‘traditional self-organised community provision’ (848–50). The objective of this study is to develop a process of engaging stakeholders in the use of clinical and research data to co-produce potential solutions, informed by a LHS, to improve long-term care for stroke survivors with multimorbidity.

## Methods

We used a stakeholder engagement study design informed by co-production principles to engage a range of stakeholders, including service users, carers, GPs and other health and social care professionals, service managers, commissioners of services, policy makers, third sector representatives and researchers working in stroke care. Our study drew on the co-production principles of equal partnership working and collaboration, reciprocity and mutuality between users and providers of services [45], using stakeholder group meetings, focus groups and nominal group techniques, as well as interviews with professionals unable to attend. Ethical approval was granted for this study by the National Research Ethics Service (NRES) Committee North East-Tyne and Wear South (REC reference number: 14/NE/1149).

### Recruitment strategy

Thirty-seven stakeholders were purposively sampled, to include stroke survivors (i.e. men and women with a range of disabilities and other long-term conditions and length of time since the stroke), family carers and professionals involved in delivering all types of stroke care and support. Participants were initially eligible to take part in the study if they were able to attend stakeholder group meetings, and were willing to provide their informed written consent. Stroke survivors were recruited through face-to-face meetings with King’s College London’s (KCL) Stroke Research Patients and Family Group (SRPFG) http://www.kcl.ac.uk/lsm/research/divisions/hscr/research/groups/stroke/forpatientsandfamily/patientsandfamily.aspx), an established advisory group of stroke surviors and carers with an interest in research. The SRPFG has a core membership of 32 stroke survivors and carers from diverse socio-economic and ethnic backgrounds and is experienced in identifying research priorities and critiquing research proposals from a service user perspective [50]. Carers and professionals were recruited respectively through the authors’ established links with voluntary sector providers, and networks of clinical, commissioning, policy and research professionals, and were invited to take part in the study via email. All participants were provided with an information sheet outlining the aim and nature of the study before providing their written informed consent to take part. The range of stakeholders and types of activity they participated in as part of this stakeholder engagement study are shown in Table 1.

**Table 1:** Stakeholders taking part in the study.

Type of stakeholder	Number (N = 37*)
**First stakeholder group meeting**	
Stroke survivor	10
Carer	1
GP (academic/clinical)	2
Physiotherapist	2
Speech and language therapist	1
Social care professional	1
Public health doctor	1
Consultant psychiatrist	1
Director of a national stroke charity	1
Policy maker	1
Commissioner	1
Service manager of national stroke charity	1
Strategic operations manager	1
**Second stakeholder group meeting**	
Stroke survivor	2
Carer	1
Physiotherapist/academic	1
Occupational therapist	1
Commissioner	1
Policy maker	1
GP (academic/clinical)	1
Postdoctoral researchers (social scientist; researchers working with SLSR/LDN dataset)	4
**Face-to-face interviews**	
GP (clinical)	1
GP and commissioner	1
GP (academic/clinical)	4
Acute stroke care nurse consultant	1
Acute stroke care consultant	1
Commissioner	1
**Third stakeholder group meeting**	
GP and commissioner	1
Stroke survivor	2
Carer	1
Commissioner	1
Occupational therapist	1
GP (academic/clinical)	1
Postdoctoral researchers (social scientist; researcher working with SLSR/LDN dataset; researcher in human factors)	3

* Note that overall 37 participants took part but a number of stakeholder representatives took part in multiple meetings

### Methods and process of stakeholder engagement

The engagement process occurred over a 10 month period and entailed stakeholder group meetings and focus groups, including the use of nominal group techniques priority setting and consensus building, which took place at the host hospital and university, and individual face-to-face interviews which took place in either the host university, GP practices, or in a quiet room in or near a hospital setting. All group meetings and interviews were audio-recorded with participants’ consent.

In the initial stakeholder group meeting, participants were introduced to the concept of a LHS and asked to identify priorities in long-term stroke care and solutions that might be derived from data capture, transformation and dissemination as the LHS proposes. This involved an introduction to the purpose of the study by the group facilitator (ES), who was a social scientist working in health research with a clinical background in physiotherapy. This was followed by focus group discussions guided by a topic guide informed by the existing literature on long-term needs and care after stroke with: 1) service users and carers; 2) health and social care professionals; and 3) commissioners, policy makers and service managers to identify stakeholder priorities. Each focus group had a facilitator, moderator and note-taker, with the latter taking notes of the main points raised, including the nature of the social interaction. Individual group priorities for needs for data and information, and potential solutions were recorded on A5 cards. The three small groups came back together as one larger group for further priority setting and consensus building. Written cards were visually displayed on flip charts. The facilitator then asked participants to prioritise identified needs for data and information and related potential solutions to improve long-term care for stroke survivors with multimorbidity.

A smaller core stakeholder group was then established to work closely, and in an on-going way, with the research team to co-produce potential interventions using available data. This consisted of service user, GP, other health care professional, policy maker and commissioner representatives, and members of the research team. The core group met and took part in a focus group discussion to consider the data available for the local stroke population that might support novel interventions to meet the previously identified priorities. These were written on a flip chart by an assistant to stimulate group discussion. A note-taker was also present at this meeting.

Since GPs and hospital clinicians were unable to attend the meetings due to clinical commitments, three authors (ES, TP, IM) conducted face-to-face individual interviews with these clinicians, using the same topic guide, to ascertain their views on priorites for data to inform potential solutions. Interviews lasted between 30–60 minutes.

Subsequently, the research team developed a number of preliminary intervention designs which could be potentially integrated as part of a LHS. A third focus group meeting with the core stakeholder group was then held to seek their feedback on the proposed interventions. Preliminary intervention designs were presented to the group and a power point presentation used to prompt discussion and consensus building, with a note-taker also present at this meeting. Taking into account stakeholder feedback, the evidence base from published literature and assessment of data availability, the research team developed a model for a data-driven intervention that was presented and discussed in a subsequent core group stakeholder meeting (which is not the focus of this paper).

### Data analysis

All qualitative data from group meetings and interviews were transcribed in full, stored in NVivo (Version 11). A thematic analysis approach was used to identify themes and subthemes [51] related to stakeholders’ priority needs for data and information, and potential interventions to improve long-term stroke care for stroke survivors with multimorbidity, noting similarities and differences between groups emerging from group meetings and interviews. This involved two authors (ES, TP) assigning codes and developing and refining themes and subthemes from the interview data.

## Results

Stakeholders prioritised four main ways in which data, or information more generally, could support improvements in long-term care for stroke survivors with multimorbidity: 1) improving continuity of care; 2) improving management of mental health consequences; 3) better access to health and social care; and 4) targeting multiple risk factors.

### Improving continuity of care

Service users/carer, providers and commissioners prioritised the need for better information in general to improve continuity of care. GPs in particular wanted improved information on interventions that had been provided in specialist care to enable a more effective transition of care from secondary to primary care, as one GP said:

‘The transition between hospital and general practice is the big area for problems. So what are the interventions that are going to make a big difference to the patient after the stroke, and to what extent have they been completed during the hospital admission? Are they being carried on afterwards under the direction of the stroke unit, or are they being transferred to the care that they get in general practice…I think there is probably a lack of clarity about the transition point.’

(GP5, interview)

Health and social care professionals and commissioners participating in the initial stakeholder group meeting also prioritised better use of integrated patient health and social care data to enable continuity of care. Professionals raised concerns about data sharing between health and social care, as National Health Service (NHS) data is highly protected and patient information would not be made freely available to third sector organisations.

In the inital stakeholder meeting, in a focus group with service users and the one carer in the group, participants emphasised the lack of information provided to manage long-term needs following hospital discharge, rather than identifying how improved use of data would enable continuity of care. Most had expected their GP to coordinate their long-term care and to provide the necessary information. However, they spoke of the perceived lack of knowledge of stroke and its long-term consequences among GPs, often sharing their experiences of limited or lack of follow-up. For example, the one carer said:

‘I don’t think she (the GP) even read, she didn’t have a copy, or made a copy of the amount of information that we were given. She viewed absolutely nothing; it was up to us to decide did we want to see a neurologist? We never had a follow-up appointment with a neurologist.’

(carer, stakeholder group meeting 1)

All stakeholders suggested general solutions only in terms of how improved use of information could enable continuity of care and interventions to support improvements in long-term care, indicating the challenges in understanding the concept and ethos of a LHS informing data-driven interventions. A number of stroke survivors proposed a checklist that GPs could use to ask patients about long-term problems related to the stroke and other health conditions:

‘We need a checklist. I have to ask my GP to refer me to a memory clinic. If they had a checklist every time they see you, they would say ‘how is your speech?’, ‘how is your memory?’. They would ask about all possible conditions.’

(stroke survivor, stakeholder group meeting 1)

Along similar lines, medical professionals suggested online patient vignettes as a proposed intervention to enable sharing of information related to optimal care and management of patients at different stages of recovery after stroke:

‘The suggestion earlier having it as a patient story is an interesting idea and using the patient’s story to see at which stage you share what type of information. So at the stage where they have just come out of hospital it might be about sharing information about best management. Then later on it might be sharing information about social support and psychological support.’

(public health doctor, stakeholder group meeting 1)

However, implementing such a ‘staged’ approach to improve long-term stroke care was received negatively among community rehabilitation therapists and one social care professional, who felt that such an approach ran counter to using a more patient-centred approach:

‘We have a very patient-centred approach and this sort of staging of a patient’s recovery does not fit at all…talking to them [patients and their family] as an individual and listening to them and looking at their context now and then rather than generalising that you have these many past medical conditions…On top of it bearing in mind their culture and other relationships, their carers’ names, their whole interaction. It is much more complex.’

(physiotherapist, stakeholder group meeting 1)

Thus the first priority stakeholders agreed on was for improved continuity of care, including effective transition of care and follow-up. However, the potential solutions they proposed focused on information provision, such as the use of a checklist, rather than how data as part of a LHS could improve continuity of care after stroke.

### Improving management of mental health consequences

Service users and professionals identified mental health consequences as a priority that needed to be addressed after stroke, particularly in terms of depression, anxiety and cognitive impairment, with improved use of data required to support potential interventions. Several service users taking part in a focus group in the initial stakeholder group meeting reported experiencing depression following their stroke, for which they received limited or no professional support. For example, one stroke survivor compared the positive care and support she had received following heart surgery with the perceived lack of support after her stroke:

‘I found after the stroke, both hospital and GP help was almost non-existent. The year before I had had a triple bypass and valve replacement, and the heart people were incredibly helpful. After the stroke I had depression, and the heart people took me on board and gave me a clinical psychologist that I could not get to as a stroke patient.’

(stroke survivor, stakeholder group meeting 1)

Professionals agreed on the need to improve the management of mental health after stroke, with several expressing the view that this was for some stroke survivors a more significant issue over time than the stroke itself. For example, the one social care professional commented:

‘People do have multiple conditions, you know. I see stroke survivors that have had mental health problems and that actually is the primary problem. They are recovering very well from their stroke and actually the main concern is their mental health.’

(social care professional, stakeholder group meeting 1)

Half of GPs interviewed concurred that a more systematic and effective approach to managing the mental health consequences of stroke, in particular depression, was a high priority to address. They proposed that improved use of data could inform a potential online screening tool to assess all stroke survivors for depression as part of routine follow-up care and management. For example, one GP said:

‘A screening tool (for depression) and of course it’s not in the QOF (Quality Outcomes Framework) is it? So it’s not there and depression screening was there for diabetes and coronary heart disease and then they took it out, much to my annoyance because I think it’s the same, similar argument but really all diabetics should be screened, all people with heart disease should be screened, certainly all people with stroke should be screened for depression.’

(GP2, interview)

In summary, a second priority identified by half of GP stakeholders was the need for a more systematic approach to managing mental health consequences after stroke, in particular depression. They proposed that improved use of data could potentially inform an online screening tool for depression. Service users reported inadequate information in general and services for follow-up for mental health problems after stroke.

### Better access to health and social care

All stakeholders largely prioritised the need for information more generally to enable better access to health and social care to address long-term needs after stroke. While stroke survivors and carers wanted improved information on access to health and social care following discharge from hospital, they often referred to procedural information they wanted from health professionals. In terms of access to health care, they spoke in particular about the need for clearer information from their GP about how to access further rehabilitation and follow-up care to address ongoing health problems, including mobility, speech and fatigue problems following their stroke.

Different stakeholders prioritised the need for information to improve access to social care to address long-term needs. Service users expressed a need for better information more generally about services to address financial problems, accessing benefits and peer support. Commissioners, service managers and policy makers, on the other hand, spoke about the lack of data on available services to alleviate social isolation after stroke, whereas health and social care professionals wanted better information on available vocational and transport support for stroke survivors, and better access to social care. Across different stakeholder groups, including among most GPs, there was consensus that a range of local community services exist but these were ‘hidden’, difficult to know about and access, or not always appropriate to meet individual needs:

‘There are just so many community services and some would be suitable for that person but not for that person and it just depends on the individual.’

(speech and language therapist, stakeholder group meeting 1)

All stakeholder groups agreed that improved information on access to health and social care could potentially support the development of an online up-to-date directory of local services with relevant eligibility criteria, organised, for example, according to postcode:

‘Why, isn’t there a system that we can all look at, you know one person knows about this and one person knows about that. Why don’t we have one system that everyone can look at, it sounds like this could potentially be it.’

(stroke survivor, stakeholder group meeting 1)

‘I think what we’re often lacking is the community services, and we could do with a really easy up-to-date directory of all the community services that are available and voluntary services because there’s obviously quite alot and they change and it’s difficult to keep up-to- date…It is difficult to stay on top of all that so that would be very useful information.’

(GP4, interview)

In summary, a third priority identified by stakeholders was for improved information about existing health and social care services. Here it was clear that stakeholders understood information on a general level, finding it difficult to imagine LHS data solutions.

### Targeting multiple risk factors

Most GPs and the one stroke physician identified the potential for data to be used to target multiple risk factors. They highlighted variations in the management of atrial fibrillation (AF) after stroke, which has been reported in the existing literature [52]. Several GPs proposed that improved use of data could potentially inform a decision support tool to optimise multiple medication management. For example, one GP said:

‘There are guidelines for each, single condition but it becomes incredibly complicated once you get two conditions, three conditions, four conditions together and you’ve got people on 8 medications, 10 medications, something like that, it then becomes, it goes up exponentially in the complexity, but there’s very little help for that in terms of guidelines and advice on what to do and you immediately hit problems with potential drug interactions and it’s very difficult to try and hold in your head potential drug interactions. That's one big area where you really do need sort of computer prompting.’

(GP2, interview)

Secondly, doctors also prioritised the need for data to identify stroke survivors at high risk of readmission to hospital, and to reduce multiple risks associated with stroke recurrence. For example, the hospital stroke physician suggested that using this information could inform a further decision support tool with computer prompting, which would have the potential to calculate risk factor scores to improve secondary prevention and management of stroke recurrence:

‘You could in a way try and identify the risk of recurrent stroke for that patient so you could have your stroke patient, you can amalgamate all your risk factors and say ‘the calculated five year risk for this patient is 20%, have you considered the following risk factor interventions?’ …So that could be a useful tool, and that’s really important because patients commonly ask us ‘what is the risk of me having another stroke in the next year’ and we come up with a figure and we say ‘5% of whatever.”

(hospital stroke physician, interview)

Management of risks also reflected concerns raised by stroke survivors about multiple medications and unwanted side effects, although this was expressed as a desire for clearer information more generally from GPs about these effects.

In summary, a fourth priority identified by stakeholders focused on the use of data to target multiple risk factors after stroke. Doctors proposed that improved use of data on multiple risk factors could potentially inform decision support tools with computer prompts to optimise medication management for stroke survivors with multimorbidity, identify those at risk of hospital readmission, and to reduce the risk of stroke reccurence.

### Preliminary intervention design solutions

To address stakeholder priorities for improved use of clinical and research data, potential interventions that could be integrated as part of a LHS were developed by the research team. These were an online depression assessment tool; an online directory of health and social care services; a decision support tool to identify patients at high risk of readmission for stroke; and a decision support tool to improve secondary prevention after stroke.

These proposals were then considered in a third core stakeholder group meeting. The first three proposals were rejected by stakeholders for a variety of reasons. The proposed tool to assess depression was considered too simplistic and it was agreed that depression was being managed more systematically in primary care since the introduction of holistic assessments and annual stroke reviews. The online directory of health and social care services presented practical problems (for example, how to ensure it was up-to-date) and would duplicate existing resources. In any case, this proposed tool would not use the linked dataset since this contained limited social care and support data. The proposed decision support tool to identify patients at high risk of readmission for stroke was also rejected since there was consensus among stakeholders that it was difficult to identify factors influencing hospital readmission after stroke.

The core stakeholder group agreed to prioritise a decision support tool to improve secondary prevention after stroke for further development, since there was a gap in evidence for an intervention targeting stroke survivors with multimorbidity, and local service provision, and relevant data were available from the linked dataset. The proposed intervention would identify stroke survivors at high risk for a recurrent stroke, display the stroke risk in an effective way for both clinicians and patients to enhance shared decision making, and propose the optimal care pathway to reduce the stroke survivor’s risk factors. Proposed outcomes would include clinical and quality of life outcomes. The group suggested that the next step would be to revise the tool based on their feedback and evaluate it with a small group of health care professionals and stroke survivors for acceptability and usability.

## Discussion

In this paper we have reported on the development and execution of a model of stakeholder engagement, informed by co-production principles, to identify and priortise novel interventions that utilise clinical and research data as part of a LHS to improve long-term care for stroke survivors with multimorbidity. Overall, stakeholders found the concept of a LHS abstract and difficult to understand in terms of how the use of data might inform interventions to improve long-term stroke care. They prioritised four main ways in which data, or information more generally, could support improvements in long-term care for stroke survivors with multimorbidity: 1) improving continuity of care; 2) improving management of mental health consequences; 3) better access to health and social care; and 4) targeting multiple risk factors. These are known consequences of long-term stroke care from the existing literature [4–11]. Based on stakeholder group consensus, consideration of the gap in evidence, current local service provision, and availability of data from the linked dataset, a decision support tool to improve secondary prevention after stroke was prioritised. Interventions addressing secondary prevention after stroke have been identified as a priority elsewhere, based on consensus opinion of stroke survivors, carers and health care professionals [53]. The stakeholder engagement process provided a platform for users and providers of stroke services to agree on priorities for service improvement and to consider ways in which clinical and research data could inform the development of potential co-produced interventions to improve long-term stroke care.

Stakeholder engagement approaches have been used to improve stroke care in other studies, for example, in developing stroke pathways [45] and co-designing tools to assess a range of long-term needs after stroke [54]. A limited number of studies have engaged a range of stakeholders to develop interventions as part of a LHS to improve health care for people with other long-term conditions, such as inflammatory bowel disease [38] and depression [39]. Heaton et al [45] drew on a co-production framework as part of a realist evaluation [55] to examine the impact of redesigning the emergency pathway after stroke on reducing the time from stroke onset to treatment. Based on analysis of interviews with clinicians and researchers they found that mechanisms of closer collaboration between stakeholders for successful knowledge translation were closely related to the core principles of co-production. We used methods as part of a stakeholder engagement study to promote key co-production principles identified by Heaton et al [45] from the literature, namely equal partnership working and collaboration, reciprocity and mutuality between service users and professionals.

### Strengths and limitations of the study

Our stakeholder engagement study adds to the co-production literature in terms of reflecting on the benefits and methodological challenges of undertaking research informed by a co-production approach to improve health care, which has received little attention [45, 46]. A strength of our study was the engagement of a range of stakeholders in a flexible manner, in which some stakeholders participated in group meetings and some were interviewed. Flexibility was needed since it was difficult to engage certain stakeholders over others in our study, which has also been reported elsewhere [46]. In particular, we found it was difficult to recruit GPs and clinicians working in acute stroke care to take part in stakeholder engagement group meetings. This led to the decision to conduct subsequent face-to-face interviews with these stakeholder groups at or near their place of work.

A more difficult problem relates to the introduction and translation of apparently complex concepts related to a LHS, to a diverse group participating in a stakeholder engagement study and co-production activities. Achieving a shared understanding of the concept of a LHS among stakeholders was difficult, especially in terms of how improved use of clinical and research data from a linked dataset as part of a LHS could potentially inform interventions to support long-term stroke care. This led to limited ‘buy in’ from stakeholders taking part in the initial stakeholder group meeting to participate in subsequent smaller core stakeholder group meetings, especially among service users and health and social care professionals working in long-term stroke care.

Furthermore, at the initial stakeholder group meeting we decided to conduct separate focus groups with different stakeholder groups followed by a larger group discussion with all stakeholders. This made group discussions more manageable and aimed to address existing knowledge hierarchies among service users and professionals. The emphasis on problem identification among stakeholders at the initial meeting lent itself well to the use of co-production methods based on nominal group techniques. Subsequent meetings with a smaller core stakeholder group to co-produce potential interventions to support improvements in long-term stroke care as part of a LHS resembled more of a stakeholder consultation style, which according to Bovaird’s model represents one of a number of different forms of co-production [49]. This raises the issue of the ‘slippery concept’ of co-production [48] in the context of conducting a stakeholder engagement study, with the current lack of consensus on what co-production is, and the methods used in practice. As a result, the boundaries of co-production research remain fluid and diffuse. Thus a tension in our study existed between the philosophy of co-production and applying this approach in practice in an applied health research context. Future research is needed to improve clarity surrounding what co-production is, and which methods are best to use in stakeholder engagement studies to improve healthcare in different contexts.

## Conclusions

We conducted a stakeholder engagement study informed by co-production principles to identify and propose data-driven interventions, informed by a LHS, that would address the long-term consequences of stroke. This process developed potential solutions and pragmatically prioritised those solutions in collaboration with users and providers of stroke services. Further work is required to evaluate the impact and implementation of co-produced data-driven interventions for long-term stroke survivors.

## Supporting information

S1 File Consolidated criteria for reporting qualitative studies (COREQ): 32-item checklist. (DOC)
