# Supplementary material for: Shaping innovations in long-term care for stroke survivors with multimorbidity through stakeholder engagement
Source: PLoS One. 2017 May 5;12(5):e0177102. doi: 10.1371/journal.pone.0177102 (PMC5419597; doi:10.1371/journal.pone.0177102)
Supplement: S1 File — (DOC) [file pone.0177102.s001.doc]

**Supplementary file 1**

Consolidated criteria for reporting qualitative studies (COREQ): 32-item checklist

| **No** | **Item** | **Guide questions/description** |
| --- | --- | --- |
| **Domain 1: Research team and reflexivity** |  |  |
| Personal Characteristics |  |  |
| 1. | Interviewer/facilitator | Which author/s conducted the interview or focus group? ES, TP, IM (reported on page 9 in the methods). |
| 2. | Credentials | What were the researcher's credentials? *E.g. PhD, MD*  ES, TP, UY, VC, CDAW, CM: PhD; IM: MD. |
| 3. | Occupation | What was their occupation at the time of the study? ES (lead author) and TP were research fellows; IM was a clinical academic fellow. |
| 4. | Gender | Was the researcher male or female?  Male and female researchers (as reported on page 9 in the methods). |
| 5. | Experience and training | What experience or training did the researcher have?  Doctoral and post-doctoral research experience of conducting qualitative research and stakeholder engagement in health research (ES, TP, IM, CM). |
| Relationship with participants |  |  |
| 6. | Relationship established | Was a relationship established prior to study commencement?  Yes, stroke survivors were recruited through an established advisory group in the authors’ host university, and carers and professionals were recruited through the authors’ established professional links (as reported on page 7 in the methods). |
| 7. | Participant knowledge of the interviewer | What did the participants know about the researcher? e*.g. personal goals, reasons for doing the research*  We state on page 7 in the methods that ‘All participants were provided with an information sheet outlining the aim and nature of the study before providing their written informed consent to take part.’ |
| 8. | Interviewer characteristics | What characteristics were reported about the interviewer/facilitator? e.g. *Bias, assumptions, reasons and interests in the research topic*  The facilitator (ES) was a social scientist working in applied health research with a clinical background in physiotherapy (as reported on page 9 in the methods). |
| **Domain 2: study design** |  |  |
| Theoretical framework |  |  |
| 9. | Methodological orientation and Theory | What methodological orientation was stated to underpin the study? *e.g. grounded theory, discourse analysis, ethnography, phenomenology, content analysis.*  A stakeholder engagement study design informed by co-production principles (as reported on page 7 in the methods). |
| Participant selection |  |  |
| 10. | Sampling | How were participants selected? *e.g. purposive, convenience, consecutive, snowball*  Participants were recruited purposively (as reported on page 7 in the methods). |
| 11. | Method of approach | How were participants approached? e*.g. face-to-face, telephone, mail, email*  Participants were approached and recruited face-to-face (stroke survivors) and via email (carers and professionals) (as reported on page 7 in the methods). |
| 12. | Sample size | How many participants were in the study?  37 participants took part in the study (as reported on page 7 in the methods and in Table 1 on page 8). |
| 13. | Non-participation | How many people refused to participate or dropped out? Reasons?  This is not reported in the paper. In the discussion on page 17 we discuss that one of the limitations of the study was ‘limited ‘buy in’ from stakeholders taking part in the initial stakeholder group meeting to participate in subsequent smaller core stakeholder group meetings’ and provide an explanation why this was the case. |
| Setting |  |  |
| 14. | Setting of data collection | Where was the data collected? e*.g. home, clinic, workplace*  Stakeholder group meetings and focus groups took place at the host hospital and university, and individual interviews took place either at the host university, in GP practices, or in a quiet room in or near a hospital setting (as reported on pages 8-9 in the methods). |
| 15. | Presence of non-participants | Was anyone else present besides the participants and researchers?  Yes, moderators and note-takers were present at the initial stakeholder group meeting and focus groups, and a note-taker was present at subsequent smaller core stakeholder groups (as reported on pages 9-10 in the methods). |
| 16. | Description of sample | What are the important characteristics of the sample? *e.g. demographic data, date.*  Type of stakeholder (as reported in Table 1 on page 8). |
| Data collection |  |  |
| 17. | Interview guide | Were questions, prompts, guides provided by the authors? Was it pilot tested?  Yes, focus groups at the initial stakeholder group meeting were ‘guided by a topic guide informed by the existing literature on long-term needs and care after stroke’ (as reported on page 9 in the methods) as were the face-to-face interviews (also reported on page 9 in the methods). |
| 18. | Repeat interviews | Were repeat interviews carried out? No |
| 19. | Audio/visual recording | Did the research use audio or visual recording to collect the data?  Yes, all group meetings and interviews were audio-recorded with participant consent (as reported on page 9 in the methods). |
| 20. | Field notes | Were field notes made during and/or after the interview or focus group?  Yes, as reported on page 9 in the methods, the note-taker at each focus group at the initial stakeholder group meeting took notes ‘of the main points raised, including the nature of the social interaction.’ |
| 21. | Duration | What was the duration of the interviews or focus group?  Interviews lasted between 30-60 minutes (as reported on page 9 in the methods). |
| 22. | Data saturation | Was data saturation discussed? No |
| 23. | Transcripts returned | Were transcripts returned to participants for comment and/or correction? No |
| **Domain 3: analysis and findings** |  |  |
| Data analysis |  |  |
| 24. | Number of data coders | How many data coders coded the data?  Two data coders (ES, TP) coded the qualitative data (as reported on page 10 in the methods). |
| 25. | Description of the coding tree | Did authors provide a description of the coding tree?  No. We discuss on page 10 in the methods that we used a thematic analysis approach to code the qualitative data and describe the steps and tools involved to systematically analyse the data. |
| 26. | Derivation of themes | Were themes identified in advance or derived from the data?  Themes and subthemes were identified from ‘stakeholders’ priority needs for data and information, and potential interventions, noting similarities and differences between groups emerging from group meetings and interviews’ (as stated on page 10 in the methods). |
| 27. | Software | What software, if applicable, was used to manage the data?  NVivo (Version X8) (as reported on page 10 in the methods). |
| 28. | Participant checking | Did participants provide feedback on the findings?  No. |
| Reporting |  |  |
| 29. | Quotations presented | Were participant quotations presented to illustrate the themes / findings? Was each quotation identified? e*.g. participant number*  Yes (as reported in the results on pages 10-15). |
| 30. | Data and findings consistent | Was there consistency between the data presented and the findings?  Yes |
| 31. | Clarity of major themes | Were major themes clearly presented in the findings?  Yes |
| 32. | Clarity of minor themes | Is there a description of diverse cases or discussion of minor themes?  Yes |
